# Clinical effects of arthroscopic-assisted uni-portal spinal surgery and unilateral bi-portal endoscopy on unilateral laminotomy for bilateral decompression in patients with lumbar spinal stenosis: a retrospective cohort study

**DOI:** 10.1186/s13018-024-04621-2

**Published:** 2024-03-05

**Authors:** Fang Wang, Rui Wang, Chengyi Zhang, En Song, Fengtao Li

**Affiliations:** 1 Department of Orthopaedics, The Second Affiliated Hospital, School of Medicine, Xi’an Jiaotong University, Xi’an, China; 2 Department of Sports Medicine, The First Affiliated Hospital of Kunming Medical University, Kunming, China

**Keywords:** Clinical effects, Endoscopic surgery, Lumbar spinal stenosis, CT imaging parameters

## Abstract

**Objective:**

To investigate the clinical effectiveness of Arthroscopic-assisted Uni-portal Spinal Surgery (AUSS) in the treatment of lumbar spinal stenosis.

**Methods:**

A total of 475 patients with lumbar spinal stenosis from January 2019 to January 2023 were included in this study. Among them, 240 patients were treated with AUSS (AUSS group); the other 235 patients were treated with unilateral bi-portal endoscopy treatment (UBE group). The differences in surgery-related clinical indicators, pain degree before and after surgery, Oswestry Disability Index (ODI), CT imaging parameters of spinal stenosis, and clinical efficacy were compared between the two groups.

**Results:**

Patients in the AUSS group had a shorter operative time than those in the UBE group, and the length of incision and surgical bleeding were less than those in the UBE group, with statistically significant differences (*P* < 0.05). Before operation, there was no significant difference in the VAS score of low back pain and leg pain between the two groups (*P* > 0. 05). After operation, patients in both groups showed a significant reduction in low back and leg pain, and their VAS scores were significantly lower than before the operation (*P* < 0.05). Three months after surgery, the results of CT re-examination in both groups showed that the spinal stenosis of the patients was well improved, and the measurements of lumbar spinal interspace APDC, CAC, ICA, CAD and LAC were significantly higher than those before surgery (*P* < 0. 05). Besides, the lumbar function of patients improved significantly in both groups, and ODI measurements were significantly lower than those before surgery (*P* < 0.05).

**Conclusion:**

Both AUSS and UBE with unilateral laminotomy for bilateral decompression can achieve good clinical results in the treatment of lumbar spinal stenosis, but the former has the advantages of simpler operation, shorter operation time, shorter incision length, and less surgical blood loss.

## Introduction

Lumbar spinal stenosis (LSS) is a disease caused by various factors such as fibrous tissue proliferation, hypertrophy of the ligamentum flavum, or osteophytes, which shortens the sagittal diameter of the spinal canal or neural root canal, resulting in neural root compression. It is mainly manifested as low back and leg pain, intermittent claudication, which seriously affect the walking ability of elderly patients, causing great pain to patients [1, 2]. In addition, elderly patients are often accompanied by some internal *medicine* diseases such as hypertension, cardiopulmonary dysfunction, and diabetes. These patients often have poor results after conservative treatment and have to choose surgical treatment [3].

In the past, open surgery was often used in the clinic, but it may excessively strip the paraspinal muscles, leading to heavy bleeding and a higher probability of postoperative lumbar instability [4, 5]. In recent years, with the rapid development of spinal endoscopy technology, it has been favoured by clinicians and patients for its advantages of small incisions, less bleeding, clear vision, and reliable decompression effect. Unilateral bi-portal endoscopy (UBE) technique, which offers both the benefits of open surgery and minimally invasive treatment, plays an important role in improving symptoms of lumbar spinal stenosis [6–9]. However, studies have shown that the rate of acquiring skills or knowledge in a short time is slower for UBE technique. During surgery, there may be situations such as instability in grasping instruments or unstable visual field [10, 11]. Our improved Arthroscopic-assisted Uni-portal Spinal Surgery (AUSS) technique is relatively simple to operate and has a wide field of view, but its use in lumbar spinal stenosis has been less studied.

Lumbar spinal stenosis is usually diagnosed or evaluated for efficacy by imaging examinations. Studies have shown that CT examination is superior in evaluating surgical effect of this disease [12]. When evaluating treatment outcomes in lumbar spinal stenosis, scholars usually use imaging examinations to measure the relevant diameter lines, but measurement of the spinal canal and dural area has been rarely reported. Besides, assessment results may also vary depending on the indicators selected.

Based on the above research background, patients with lumbar spinal stenosis were included in this study. A CT image analysis system has been used to analyse the relevant diameters and areas of the lumbar spine. Combined with conventional perioperative and postoperative evaluation indicators, this study dedicated to explore the differences between the effects of AUSS and UBE technique in spinal endoscopic decompression for lumbar spinal stenosis, aiming to provide a therapeutic basis for the clinical application of AUSS technique.

## Materials and methods

### Study design and inclusion/exclusion criteria

A total of 475 patients with lumbar spinal stenosis hospitalized from January 2019 to January 2023 were selected for this study. Patients were randomly divided using a random number table. Among them, 240 patients were treated with endoscopic spinal decompression using the AUSS technique (AUSS group); another 235 patients were treated with UBE technique (UBE group).

*Inclusion criteria*: ① The patients have typical symptoms such as low back pain, limb dysfunction, and intermittent claudication; ② patient age range: 45–79 years old; ③ CT and MRI show that the sagittal diameter of the spinal canal was less than 1.0 cm, and the lateral recess spacing was less than 0.3 cm; ④ the patient's conservative treatment was not effective and surgery was required.

*Exclusion criteria*: ① Patients with spinal tumours, tumour metastasis, ankylosing spondylitis, and spinal tuberculosis infection; ② bleeding tendencies, platelet abnormalities, or coagulation abnormalities; ③ patients with multi-segmental (≥ 3 segments) lesions; ④ patients with acute myocardial infarction, cerebrovascular disease, severe heart failure or major surgery within the past 3 months.

### Surgical technique

**Fig. 1:**
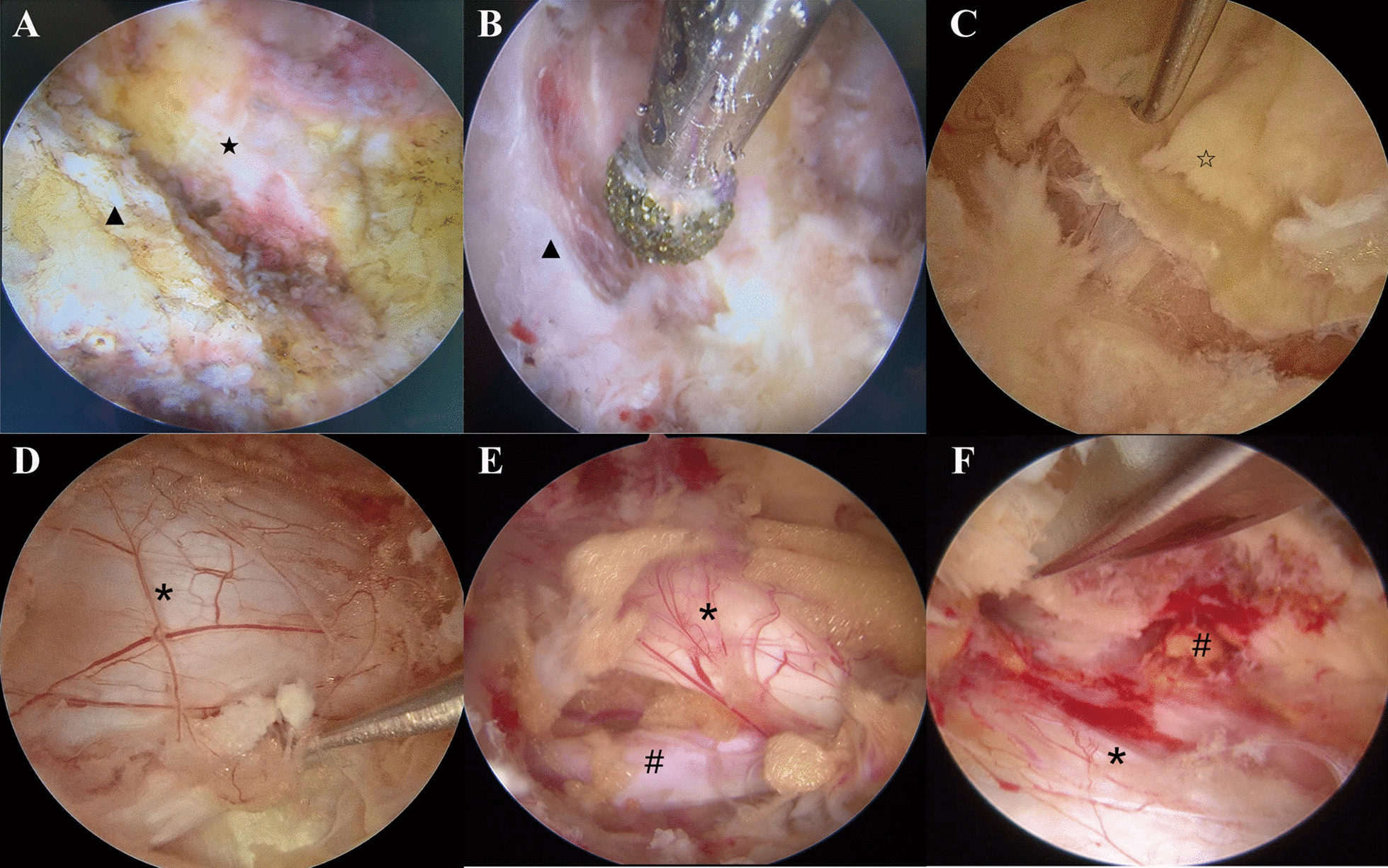
Performance of AUSS technique for bilateral decompression in the LSS patient. **A** Find the lower margin of the upper laminae and the laminae space. **B** The bone was removed in a circular manner along the lower margin of the upper laminae, the facet joints, the upper margin of the lower laminae, and the root of the spinous process. **C** Complete exposure and uncovering of the ligamentum flavum. **D** Exposure to the dural sac of the decompression segment. **E** Adequate decompression of the ipsilateral traversing nerve root and the dural sac. **F** Adequate decompression of the contralateral traversing nerve root and the dural sac. ▲, the lower margin of the upper laminae; ★, interlaminar space; ☆, ligamentum flavum;** #**, nerve root; *, dural sac

AUSS technique: AUSS refers to the single-hole split spine endoscopic technique. Parallel operation of endoscopes and instruments in a single hole. Therefore, it can also be called single-hole dual-entry endoscopic procedure. AUSS technique is based on the UBE technique, which shares a common set of instruments with UBE. AUSS combines the single-sided dual-channel double holes into a single hole, adding an endoscope to the conventional open surgery, with the advantages of open observation field, free operation space, and compatibility with a variety of spinal surgery techniques and instruments. AUSS is a continuation and upgrade of the intervertebral foraminoscopy and an improvement of the unilateral dual-channel technique. The brief steps are as follows: place the patient in a prone position and operate under general anesthesia. Determine the intervertebral space of the diseased segment under C-arm fluoroscopy. Insert a needle into the intervertebral space medial to the pedicle, move the needle into the proximal articular process on the lateral side of the vertebral plate, replace with a guidewire, and then pull out the needle. Make an incision about 1.5 cm long at the entrance of the needle, enlarge the passage by using a dilation tube, withdraw the dilation tube after dilation, and then insert a working trocar to facilitate insertion of the spinal endoscope. The difference in this plan is firstly performing laminectomy and lateral recess decompression, and then removing the proliferative ligamentum flavum to fully expose the nerve root and dural sac. Decompress along the nerve root outlet using a rongeur, enlarge the intervertebral foramen, and then check for nerve root laxity, as shown in Fig. 1. After surgery, rinse the wound, inject 2 ml betamethasone, and suture the skin.

UBE technique: preoperative preparation and localization of the intervertebral space in the diseased segment were the same as in the AUSS group. Points approximately 1 to 1.5 cm above and below the intersection of the midline of the intervertebral space and the inner edge of the pedicle were used as observation and surgical holes, respectively. An intervertebral foraminoscope was placed at the head end as an observation channel, and the caudal incision was chosen as the surgical channel to facilitate access to the decompression surgical instruments. Steps: incised sequentially the skin, subcutaneous tissue, and fascia, use a dissector to pass through the paraspinal muscles to reach the vertebral plate, and passively separate the soft tissue overlying it. At the work channel, use a radiofrequency scalpel to separate the ligamentum flavum and the soft tissue on the vertebral plate. Perform laminectomy decompression and then the remaining steps are the same as in the AUSS group.

### Observation indexes and evaluation methods

Compare the clinical indicators related to surgery, pain level before and after surgery, Oswestry disability index (ODI), CT imaging parameters of spinal stenosis including the anterior–posterior diameter of the canal (APDC), cross-sectional area of the canal (CAC), inscribed circle area (ICA), cross-sectional area in the dura (CAD), and libarea of the canal (LAC), as well as differences in clinical outcomes. The postoperative pain level of patients was evaluated using the Visual Analogue Pain Scale (VAS), which evaluated their low back and leg pain levels before, 3 months, and 6 months after the surgery. The maximum score is 10, and the minimum score is 0. The more severe the pain, the higher the score. The ODI dysfunction index can evaluate patient dysfunction, with a total of 10 items and 6 alternative answers for each item (0–5 points; 0 indicates no dysfunction and 5 indicates the most severe dysfunction) [13, 14]. The ODI value is calculated by adding the scores of each item to the total score and calculating the percentage of the highest score of the 10 items (50 points). The closer the index is to 100%, the more severe it is, and 0% is normal. Besides, we evaluated the therapeutic effect using the modified Macnab efficacy standard, which was classified as excellent, good, acceptable, and poor based on the patient's functional recovery after 6 months of surgery. Excellent: Complete disappearance of symptoms and return to work and life as usual; good: slight symptoms, slightly limitation of activity, no impact on work and life; acceptable: reduce symptoms, limited activity affecting normal work and life; poor: no difference or even worsening before and after treatment. Before and 3 months after surgery, a GE64 row CT machine was used for examination. The examination sites were L4/5 and L5/S1 intervertebral spaces, and the scanning conditions were as follows: scan 4 layers, layer thickness 5 mm, voltage 140 kV, current 284 mA, matrix: 512 × 512. The measurement indicators include ICA, CAC, APDC, LAC, and CAD. CAC: The area measured from the anterior edge of the ligamentum flavum, the inner edge of the pedicle, and the outer edge of the nerve root, with the intervertebral disc as the point. APDC: The distance from the midpoint of the posterior edge of the vertebral body to the anterior edge of the spinous process. ICA: The area of an inscribed circle drawn within the central spinal canal at the above-mentioned spinal canal boundary. LAC: Using soft tissues such as the posterior longitudinal ligament and ligamentum flavum within the spinal canal as the inner boundary, determine the area of the spinal canal surrounded by soft tissues. CAD: Through circular scanning, taking the outer boundary of the dural sac as the range, the enclosed area is called CAD. The area enclosed by CAD and CAC is called LAC.

### Statistical analyses

IBM SPSS Version 22.0 (IBM Corporation, Armonk, New York, USA) was used to perform the statistical analyses. The measurement data of APDC, CAC, ICA, CAD, and LAC in the lumbar intervertebral space collected in this study that conform to normal distribution were described using (${\overline{\text{x}}}$ ± s), and the comparison between the two groups was conducted using independent sample t tests; repetitive measurement analysis of variance was used for inter-group comparison of repeated econometric data; counting data (gender, lesion segment, disease classification, treatment effect, etc.) should be described using component ratios or rates, and comparative analysis between groups should be conducted chi-square test; rank counting data were tested using Mann–Whitney *U* test; *P* < 0.05 indicates that the difference is statistically significant.

## Results

### Comparison of preoperative baseline data

Table 1 shows the comparison of the preoperative basic data of the patients in the two groups. The difference between the two groups was not statistically significant (*P* < 0. 05), indicating that the randomized grouping is reasonable.

**Table 1:** Demographic data of the patients in AUSS and UBE groups

	AUSS	UBE	t/x^2^	*P*
Number of patients	240	235	/	/
Age	56.7 ± 7.8	57.9 ± 6.9	1.775	0.0766
Sex(male/female)	145/95	123/112	3.15	0.0759
BMI	24.3 ± 2.3	24.6 ± 2.1	1.484	0.1386
Duration of disease(months)	18.8 ± 7.8	19.4 ± 8.6	0.7968	0.426
Diseased segment				
L2/3	1	2	0.709	0.8711
L3/4	12	10		
L4/5	124	117		
L5/S1	103	106		
Type of spinal canal stenosis (Central/lateral recess/mixed)	29/38/173	40/23/172	5.393	0.0674

*AUSS* Arthroscopic-assisted Uni-portal Spinal Surgery; *UBE* unilateral bi-portal endoscopy; *BMI* body mass index

### Comparison of clinical surgical indicators

**Fig. 2:**
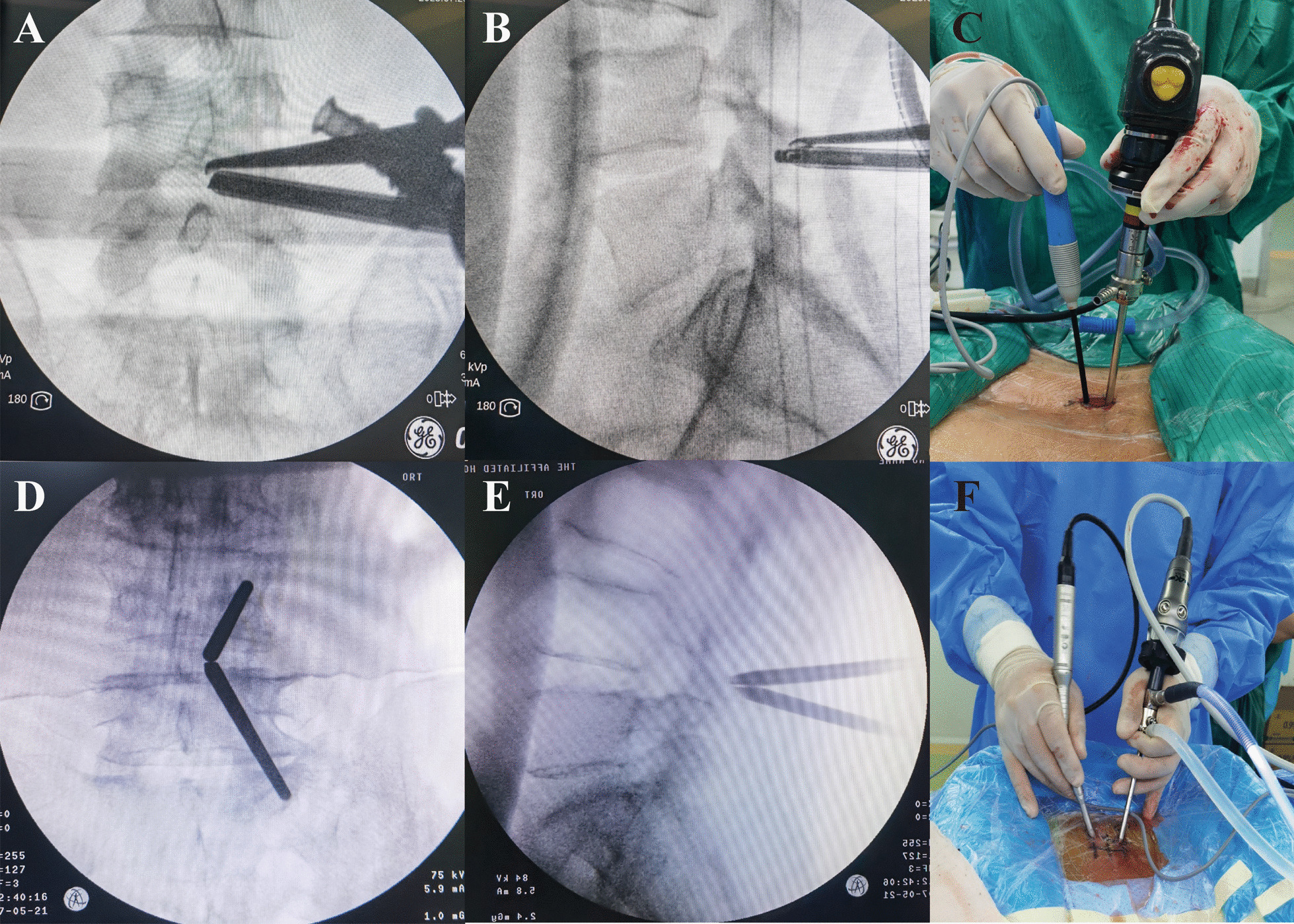
Positioning and external observation before spinal decompression were performed by the two surgical methods. **A** and **B** Target disc space by AUSS method was confirmed via posteroanterior and lateral fluoroscopy. **C** Operative photograph showing OSE method for bilateral decompression. **D** and **E** Target disc space by the UBE method was confirmed via posteroanterior and lateral fluoroscopy. **F** Operative photograph showing UBE method for bilateral decompression

Figure 2 shows the two surgical methods of intraoperative operation processes for typical cases, respectively. The surgical process and postoperative recovery of patients in the two groups were analysed. The operative time of patients in the AUSS group was shorter than that in the USE group, and the amount of surgical bleeding was less than that in the USE group (*P* < 0. 05). However, there were no significant difference between the two groups in terms of postoperative bed rest and hospital stay (*P* > 0. 05). See Table 2.

**Table 2:** Comparison of clinical surgical indicators in AUSS and UBE groups

	AUSS	UBE	*t*	*P*
Operation time (min)	50.2 ± 8.9	55.7 ± 9.3	6.585	< 0.0001
Incision length (cm)	1.5 ± 0.2	3.1 ± 0.3	68.52	< 0.0001
Intraoperative blood loss (mL)	64.7 ± 7.8	74.2 ± 6.9	14.05	< 0.0001
Time in bed (h)	16.7 ± 6.3	16.4 ± 7.4	0.4761	0.6342
Hospital stay (d)	4.8 ± 2.4	5.1 ± 2.8	1.255	0.2102

*AUSS* Arthroscopic-assisted Uni-portal Spinal Surgery; *UBE* Unilateral bi-portal endoscopy

### Comparison of low back pain scores and ODI before and after surgery

In the VAS scores of low back and leg pain, there was no statistically significant difference between the AUSS group and the UBE group (*P* > 0 05). Besides, after surgery, the pain levels in the lower back and leg of both groups of patients were significantly reduced, and the VAS score was significantly lower than before surgery (*P* < 0.05); See Table 3.

**Table 3:** Comparison of low back pain scores and ODI in AUSS and UBE groups

	AUSS	UBE	F	*P*
*Lumbar VAS score*				
pre-operation	5.6 ± 1.4	5.7 ± 1.6	*F*_interaction_ = 3.683	*P* = 0.0254
3 months after operation	2.4 ± 0.8^*^	2.6 ± 1.1^*^	*F*_time_ = 1331	*P* < 0.0001
6 months after operation	2.1 ± 1.1^*^	1.9 ± 0.9^*^	*F*_inter-group_ = 0.2833	*P* = 0.5946
*Leg VAS score*				
pre-operation	6.5 ± 1.3	6.7 ± 1.1	*F*_interaction_ = 4.132	*P* = 0.0162
3 months after operation	2.5 ± 1.2^*^	2.3 ± 1.3^*^	*F*_time_ = 2065	*P* < 0.0001
6 months after operation	2.0 ± 1.1^*^	1.8 ± 1.4^*^	*F*_inter-group_ = 1.033	*P* = 0.3096
*ODI (%)*				
pre-operation	67.8 ± 10.7	64.8 ± 11.4	*F*_interaction_ = 6.865	*P* = 0.0011
3 months after operation	18.7 ± 6.7^*^	19.6 ± 7.8^*^	*F*_time_ = 5478	*P* < 0.0001
6 months after operation	15.4 ± 5.6^*^	15.2 ± 6.2^*^	*F*_inter-group_ = 2.994	*P* = 0.0838

Preoperative comparison with this group ∗ *P* < 0. 05

*AUSS* Arthroscopic-assisted Uni-portal Spinal Surgery; *UBE* Unilateral bi-portal endoscopy; *ODI* Oswestry disability index; *VAS* Visual analogue scale

There was no statistically significant difference in ODI values between the AUSS group and the UBE group before surgery (*P* > 0 05); the postoperative lumbar function of both groups of patients was significantly improved, and the ODI measurement value was significantly lower than that of this group before surgery (*P* < 0.05). See Table 3.

### Comparison of CT imaging parameters before and after surgery

**Fig. 3:**
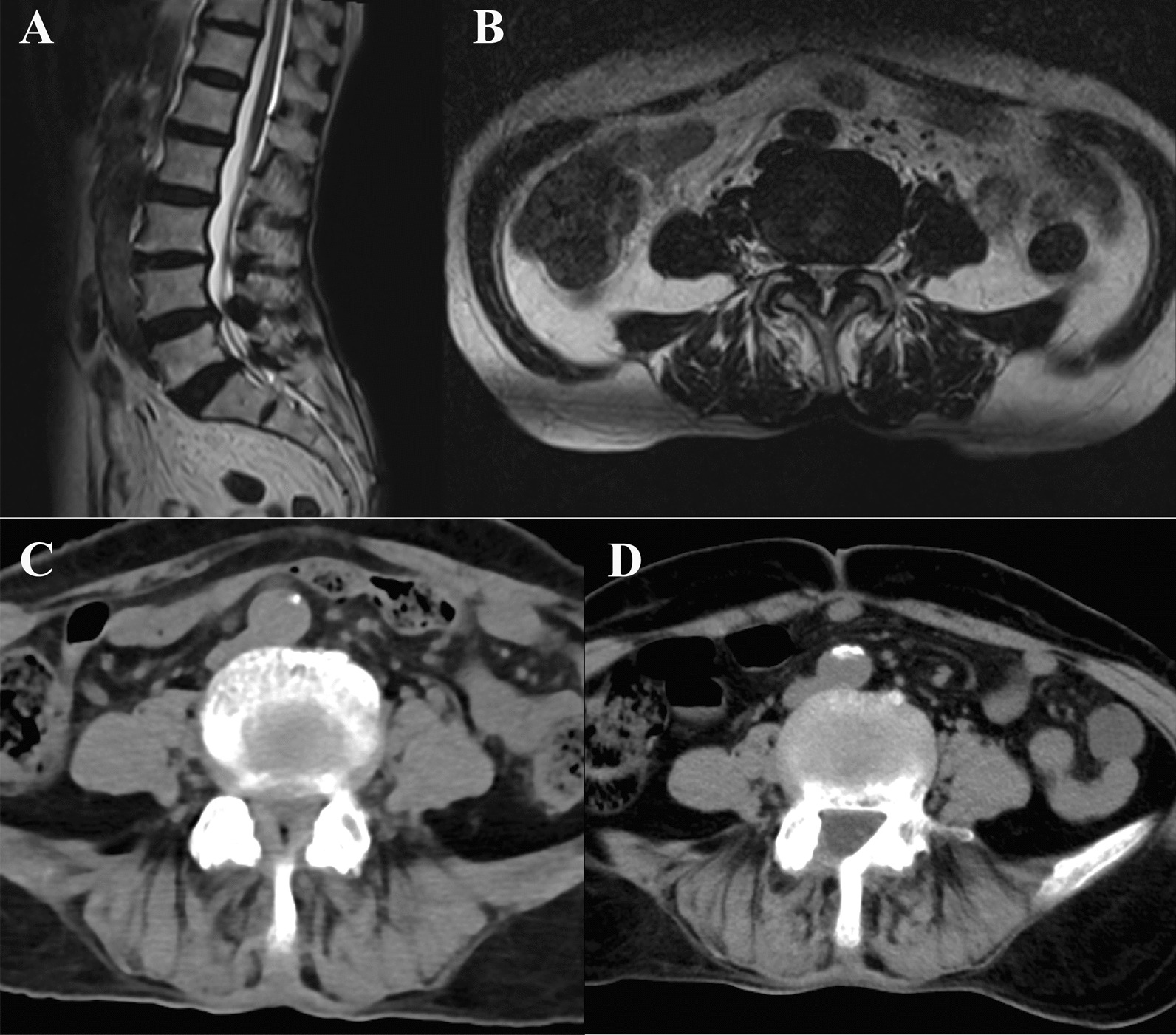
Imaging data of a typical case of L4/L5 lumbar spinal stenosis undergoing AUSS treatment. **A**, **B** Preoperative magnetic resonance imaging. **C** Preoperative computed tomography. **D** Computed tomography 1 week after surgery

Before surgery, there was no statistically significant difference in the measurement values of APDC, CAC, ICA, CAD, and LAC between the AUSS group and the UBE group (P > 0.05). Patients in the two groups underwent CT re-examination 3 months after surgery and the results show that the stenosis of the spinal canal in the patients was significantly improved. Figure 3 shows the imaging data of a typical case of L4/L5 lumbar spinal stenosis under AUSS treatment. The measurement values of APDC, CAC, ICA, CAD, and LAC were significantly increased compared to those before surgery (*P* < 0.05). Comparing the differences in lumbar interspace APDC, CAC, ICA, CAD, and LAC before and after treatment between the AUSS group and the UBE group, the differences were not statistically significant (*P* > 0 05). See Table 4.

**Table 4:** Comparison of CT imaging parameters before and after operation

	AUSS	UBE	t	*P*
APDC(mm)				
pre-operation	13.3 ± 1.6	13.5 ± 1.4	1.575	0.5215
3 months after operation	15.4 ± 1.3^*^	15.3 ± 1.2^*^	0.7875	0.9661
CAC (mm^2^)				
pre-operation	156.5 ± 28.9	153.9 ± 33.4	0.9018	0.9359
3 months after operation	314.4 ± 32.5^*^	309.3 ± 30.7^*^	1.769	0.3826
ICA (mm^2^)				
pre-operation	92.6 ± 16.7	96.4 ± 18.4	2.009	0.2403
3 months after operation	234.5 ± 23.6^*^	230.9 ± 22.9^*^	1.904	0.298
*CAD (mm*^*2*^*)*				
pre-operation	127.8 ± 32.3	133.2 ± 31.4	1.816	0.3517
3 months after operation	218.3 ± 34.2^*^	221.4 ± 31.6^*^	1.042	0.8798
*LAC (mm*^*2*^*)*				
pre-operation	14.8 ± 3.6	15.6 ± 4.1	1.749	0.3961
3 months after operation	32.7 ± 5.7^*^	33.6 ± 6.1^*^	1.968	0.2622

Preoperative comparison with this group ∗ *P* < 0. 05

*ICA* Inscribed circle area; *CAC* cross-sectional area of the canal; *APDC* Sagittal diameter of the canal; *LAC* Epidural space; *CAD* Cross-sectional area in the dura

### Treatment effect evaluation and complications

The clinical effects of the two groups were evaluated 6 months after surgery. According to the modified Macnab efficacy standard, there was no statistically significant difference in the treatment effect of spinal stenosis between the AUSS group and the UBE group (*P* > 0.05). In addition, the incidence of postoperative complications was 1.25% in the AUSS group and 2.55% in the UBE group; the difference in both groups was not statistically significant (*P* > 0. 05). See Table 5.

**Table 5:** Treatment effect evaluation and complications in AUSS and UBE group

	AUSS	UBE	X^2^	*P*
*Therapeutic effect*				
excellent	92	80	4.328	0.2282
good	120	112		
acceptable	26	39		
poor	2	4		
*Complications*				
infection	0	1	0.6	0.7408
nerve injury	2	3		
dural laceration	1	2		

## Discussion

Lumbar spinal stenosis has a huge impact on patient's life, and surgery is a good choice for patients who do not respond well to conservative treatment. According to the studies [15, 16], UBE has a large operating angle and a similar decompression effect to open surgery. It has minimal damage to the paraspinal muscles during surgery, which significantly improves the stability of the spine. Its effectiveness in treating lumbar spinal stenosis has been extensively studied and confirmed. AUSS technique is modification of the UBE technique, which can be quickly applied and significantly improve the spinal stability and patient’s mobility. However, there are few studies comparing the effectiveness of two surgical treatments for lumbar spinal stenosis. This study is the first to comprehensively analyse and compare the effects of AUSS and UBE techniques for spinal endoscopic decompression in the treatment of lumbar spinal stenosis, as well as their impacts on patients’ imaging-related indexes, perioperative indexes, and postoperative functional indexes.

The results showed that the operation time in the AUSS group was shorter than that in the UBE group, the intraoperative blood loss was less than that in the UBE group, and the length of the incision was smaller than that in the UBE group. Besides, there was no significant difference in postoperative bed rest and hospital stay between the two groups. These results indicate that the AUSS technique has more advantages than UBE in shortening the operation time, decreasing bleeding, and reducing the length of surgical incision. For the clinical therapeutic advantages of AUSS, we thought they might be attributed to the following points: (1) Compared with the UBE technique, the intraoperative localization of AUSS is simpler and facilitates rapid determination of the window-opening range. Besides, the UBE method is relatively complex, the surgical segment needs to be defined by fluoroscopy, and there is a higher chance of intraoperative instruments colliding and obstructing each other, which makes the risk of surgical bleeding greater. (2) AUSS technique uses a single hole, which greatly reduces damage to the surrounding tissues from the radio-frequency electrodes compared to UBE. When UBE strips muscle tissue, it increases damage to surrounding tissues, leading to increased bleeding [17]. (3) AUSS technique is relatively simple to decompress and has a short learning curve, making it easier for physicians to familiarize themselves with and master. In contrast, the UBE technique is a difficult procedure with a steep learning curve.

In addition, our results showed that patients in both groups experienced a significant reduction in postoperative low back and leg pain and a significant reduction in VAS scores compared to preoperative periods, suggesting that both techniques could improve the pain symptoms of the patients. UBE can technique decompress the spinal canal and protect the medial branch of the dorsal branch of the spinal nerve without additional stretching of the relevant muscles, thus improving the patient's pain. Therefore, the technology not only meets the requirements of minimally invasive but is also practical. While adhering to minimally invasive surgery, AUSS technique further expands the scope of surgical operation and decompression, avoids the removal of surrounding normal tissues during surgery, which greatly reduces the degree of damage to the surrounding tissues, and improves the postoperative pain of patients to a certain extent.

Currently, it is common to diagnose lumbar spinal stenosis or evaluate its treatment based on symptoms, signs, and imaging data [18, 19]. In this study, CT imaging software was used to measure changes in relevant regional parameters that can reflect complex anatomical morphology, which was superior to predicting postoperative outcomes by simple radial lines. On CT review at 3 months postoperatively, the measurements of APDC, CAC, ICA, CAD, and LAC in the two groups were significantly higher than those in the corresponding preoperative groups, indicating that both surgeries could significantly improve the abnormal anatomy of the lumbar stenosis site. Moreover, both procedures are performed through an interlaminar approach that provides adequate decompression of the central spinal canal and nerve root canal. Previous studies have found [20] that both methods can significantly improve lumbar scoliosis and axial rotation. Besides, some studies [21, 22] found that both surgeries not only improve the congenital instability of the lumbar spine, but also slow down lumbar degeneration. In this study, we found that both surgical techniques provided relief from the symptoms of lumbar spinal stenosis. Additionally, both of them could preserve the integrity of the facet joint, spinous processes, and supraspinous ligaments, which is important for maintaining spinal stability.

At the same time, the results of our study also showed a significant improvement in lumbar spine function in both groups, with ODI measurements significantly lower than preoperative values. The statistics of postoperative complications showed that there was no significant difference in the complication incidence between the two groups. It shows that both surgical methods have an improving effect on the dysfunction of the body, and confirms that both methods are less damaging to the paravertebral muscles, which is conducive to improving the stability of the spine. Moreover, both surgical methods can improve the pain symptoms of patients and better maintain spinal stability and motion after surgery, which is conducive to a speedy recovery and improved postoperative dysfunction.

However, this study also has some shortcomings. Cause AUSS technique is an improvement on the domestic UBE technique, the study lacks long-term follow-up data for domestic patients, and the impact on long-term prognosis is not clear enough.

In summary, both AUSS and UBE techniques can achieve good clinical results in the treatment of lumbar spinal stenosis, with the differences being that the former is simpler to perform, has a shorter operative time, a smaller incision, and causes less surgical bleeding.

## Conclusion

Compared to UBE, AUSS technique has the advantages of simpler operation, shorter operation time, shorter incision length, and less surgical blood loss.

## Data Availability

The data used to support the findings of this study are available from the first author upon request.

## References

[1] Lai MKL, Cheung PWH, Cheung JPY (2020). A systematic review of developmental lumbar spinal stenosis. Eur Spine J.

[2] Kitab S, Habboub G, Abdulkareem SB, Alimidhatti MB, Benzel E (2019). Redefining lumbar spinal stenosis as a developmental syndrome: Does age matter?. J Neurosurg Spine.

[3] Lamas V, Gueugnon M, Fournel I, Grelat M, Maillefert JF, Ornetti P, Martz P (2021). Dynamic global sagittal alignment in patients with lumbar spinal stenosis: analysis of the effects of decompression surgery on gait adaptations. Gait Posture.

[4] Kobayashi Y, Tamai K, Toyoda H, Terai H, Hoshino M, Suzuki A, Takahashi S, Hori Y, Yabu A, Nakamura H (2021). Clinical outcomes of minimally invasive posterior decompression for lumbar spinal stenosis with degenerative spondylolisthesis. Spine Phila Pa (1976).

[5] Shen J, Wang Q, Wang Y, Min N, Wang L, Wang F, Zhao M, Zhang T, Xue Q (2021). Comparison between fusion and non-fusion surgery for lumbar spinal stenosis: a meta-analysis. Adv Ther.

[6] Xu J, Wang D, Liu J, Zhu C, Bao J, Gao W, Zhang W, Pan H (2022). Learning curve and complications of unilateral biportal endoscopy: cumulative sum and risk-adjusted cumulative sum analysis. Neurospine.

[7] Park MK, Son SK, Park WW, Choi SH, Jung DY, Kim DH (2021). Unilateral biportal endoscopy for decompression of extraforaminal stenosis at the lumbosacral junction: surgical techniques and clinical outcomes. Neurospine.

[8] Chu PL, Wang T, Zheng JL, Xu CQ, Yan YJ, Ma QS, Meng-Chen Y, Da-Sheng T (2022). Global and current research trends of unilateral biportal endoscopy/biportal endoscopic spinal surgery in the treatment of lumbar degenerative diseases: a bibliometric and visualization study. Orthop Surg.

[9] Zhou H, Wang X, Chen Z, Liu W, Luo J (2023). Unilateral biportal endoscopy versus microscopic decompression in the treatment of lumbar spinal stenosis: a meta-analysis. Medicine (Baltimore).

[10] Liang J, Lian L, Liang S, Zhao H, Shu G, Chao J, Yuan C, Zhai M (2022). Efficacy and complications of unilateral biportal endoscopic spinal surgery for lumbar spinal stenosis: a meta-analysis and systematic review. World Neurosurg.

[11] Tan B, Yang QY, Fan B, Xiong C (2023). Decompression via unilateral biportal endoscopy for severe degenerative lumbar spinal stenosis: a comparative study with decompression via open discectomy. Front Neurol.

[12] Perez FA, Quinet S, Jarvik JG, Nguyen QT, Aghayev E, Jitjai D, Hwang WD, Jarvik ER, Nedeljkovic SS, Avins AL (2019). Lumbar spinal stenosis severity by CT or MRI does not predict response to epidural corticosteroid versus lidocaine injections. AJNR Am J Neuroradiol.

[13] Charalampidis A, Canizares M, Kalsi PS, Hung WuP, Johnson M, Soroceanu A, Nataraj A, Glennie A, Rasoulinejad P, Attabib N (2022). Differentiation of pain-related functional limitations in surgical patients with lumbar spinal stenosis (LSS) using the Oswestry Disability Index: a Canadian Spine Outcomes and Research Network (CSORN) study. Spine J.

[14] Jespersen AB, Gustafsson M (2018). Correlation between the Oswestry Disability Index and objective measurements of walking capacity and performance in patients with lumbar spinal stenosis: a systematic literature review. Eur Spine J.

[15] Jing X, Gong Z, Qiu X, Zhong Z, Ping Z, Hu Q (2022). "Cave-in" decompression under unilateral biportal endoscopy in a patient with upper thoracic ossification of posterior longitudinal ligament: Case report. Front Surg.

[16] Kim SK, Kang SS, Hong YH, Park SW, Lee SC (2018). Clinical comparison of unilateral biportal endoscopic technique versus open microdiscectomy for single-level lumbar discectomy: a multicenter, retrospective analysis. J Orthop Surg Res.

[17] Wang D, Xu J, Zhu C, Zhang W, Pan H (2023). Comparison of outcomes between unilateral biportal endoscopic and percutaneous posterior endoscopic cervical keyhole surgeries. Medicina Kaunas.

[18] Ammendolia C, Hofkirchner C, Plener J, Bussières A, Schneider MJ, Young JJ, Furlan AD, Stuber K, Ahmed A, Cancelliere C (2022). Non-operative treatment for lumbar spinal stenosis with neurogenic claudication: an updated systematic review. BMJ Open.

[19] Zaina F, Tomkins-Lane C, Carragee E, Negrini S (2016). Surgical versus non-surgical treatment for lumbar spinal stenosis. Cochrane Database Syst Rev.

[20] Kang MS, Heo DH, Kim HB, Chung HT (2021). Biportal endoscopic technique for transforaminal lumbar interbody fusion: review of current research. Int J Spine Surg.

[21] Kim JE, Choi DJ, Park EJ (2018). Clinical and radiological outcomes of foraminal decompression using unilateral biportal endoscopic spine surgery for lumbar foraminal stenosis. Clin Orthop Surg.

[22] Zheng B, Zhang XL, Li P (2023). Transforaminal interbody fusion using the unilateral biportal endoscopic technique compared with transforaminal lumbar interbody fusion for the treatment of lumbar spine diseases: analysis of clinical and radiological outcomes. Oper Neurosurg (Hagerstown).

